# Prevalence of Iron Deficiency Anaemia in Elderly Aged > 60 Taking Daily Low-Dose Aspirin

**DOI:** 10.1155/jare/6989003

**Published:** 2025-11-28

**Authors:** Ali A. Alaklabi, Abdulrahman S. Altowaim, Hassan S. Alqahtani, Abdulrahman A. Bin Moammar, Abdulaziz T. Alturki, Rakan A. Al Muammar

**Affiliations:** ^1^College of Medicine, King Saud Bin Abdulaziz University for Health Sciences, Riyadh, Saudi Arabia; ^2^College of Medicine, King Abdullah International Medical Research Center, Riyadh, Saudi Arabia; ^3^Department of Medicine, King Abdulaziz Medical City, Riyadh, Saudi Arabia

**Keywords:** elderly population, gastrointestinal bleeding, haemoglobin levels, iron deficiency anaemia, low-dose aspirin, retrospective cohort study

## Abstract

Iron deficiency anaemia (IDA) and anaemia of chronic disease are both common causes of morbidity in the elderly population, where they increase hospitalization and mortality. The risk of IDA is increased in gastrointestinal bleeding caused by aspirin at low doses administered daily, which has been widely used to prevent cardiovascular diseases. The current research will compare the occurrence of IDA between patients under 81 mg of low-dose aspirin and nonaspirin users. The electronic medical records were reviewed for a retrospective cohort study in the King Abdulaziz Medical City, Riyadh, Saudi Arabia (2016–2023). The inclusion criteria were patients aged over 60 years who were taking 81 mg aspirin daily, and 1308 patients were included (654 aspirin users and 654 nonusers). The number of men using aspirin was 378 (56.5), and those not using it were 291 (43.5). The number of women using aspirin was 276 (43.2), and those not using it were 363 (56.8). In the IDA cases, there were 136 (20.8) and 165 (25.2) cases in aspirin and nonaspirin users, respectively. Although this was not a significant difference, the load of haemoglobin in aspirin users was markedly less with a high concentration of ferritin in the bloodstream than in nonusers. Hypertension was a risk factor for IDA. The IDA rates were higher in nonaspirin users in females, old patients and chronic steroid users. Aspirin and nonaspirin users did not differ significantly in terms of IDA incidence. Nonetheless, there were a decreased haemoglobin and an increased ferritin in aspirin users. Potential confounders may include age and BMI, which need to be considered when monitoring low-dose aspirin patients in old age.

## 1. Introduction

In the elderly, anaemia is characterized by haemoglobin (Hb) levels below 120 g/L in women and below 130 g/L in men [1]. Anaemia prevalence affects, according to global estimates, 39% of individuals aged > 60 years; it is 54% in the Asian elderly population. (1) The two common causes of anaemia among the elderly include iron deficiency anaemia (IDA) and anaemia of chronic diseases [2]. Furthermore, anaemia is considered an independent risk factor contributing to higher rates of hospitalization, morbidity and mortality [3–5].

The high prevalence of IDA among the elderly population reflects factors such as poor dietary habits, systemic diseases and medication use [6]. Low-dose aspirin, usually taken by the elderly to prevent cardiovascular events, exerts this action through platelet aggregation inhibition. This, however, increases the risk of gastrointestinal bleeding, which is a major cause of iron loss leading to IDA. A large randomized controlled trial involving > 19,000 healthy adults over 70 years, randomized to low-dose aspirin or placebo and with an average follow-up of about four years, showed an increased incidence of upper and lower gastrointestinal haemorrhage with aspirin use. The study identified the independent risk factors as ageing, hypertension (HTN), obesity, smoking history and chronic renal disease [7]. The study further concluded that even without significant bleeding, low-dose aspirin lowered ferritin levels and increased anaemia incidence in healthy elderly people [7]. Another study concluded that the relationship between low-dose aspirin and anaemia remains unclear when there is no apparent bleeding. However, low-dose aspirin exposure and an Hb decline may be related among the elderly population [8].

As mentioned above, previous literature has estimated the risk of aspirin-associated gastrointestinal bleeding, but most studies have not explicitly focused on the elderly population or included stratification by age. Very few studies have directly compared the incidence of IDA and associated risk factors between older adults taking daily low-dose aspirin and those not taking it. The significance of this association lies in the risk profiling and calculation of the benefit-to-risk ratio for aspirin treatment, especially in the elderly, who have higher prevalence rates for both cardiovascular diseases and IDA.

Patients aged > 60 years were selected for this study as anaemia and its associated disorders are prevalent in this age group. This demographic is thus relevant when assessing the effect of low-dose aspirin (81 mg), which is used to prevent cardiovascular diseases and likely increases the risk of IDA resulting from gastrointestinal bleeding. Anaemia may be facilitated by ageing itself, through decreased gastrointestinal absorption, chronic diseases and the cumulative effects of medications. The main aim of this study is to investigate the incidence of IDA in elderly patients aged 60 and above who use daily low-dose aspirin (81 mg) and compare it with the incidence of nonaspirin users of similar age. This study would highlight the impact of low-dose aspirin (81 mg) on anaemia and provide insights to guide clinical therapeutic decisions regarding its use in elderly patients.

## 2. Materials and Methods

### 2.1. Study Design and Settings

This observational, retrospective cohort study was undertaken at King Abdulaziz Medical City, Riyadh (KAMC-RD), Saudi Arabia, to estimate the incidence of IDA in patients aged 60 years and above who use low-dose 81 mg aspirin daily. KAMC-RD began delivering services in May 1983 and offers a comprehensive range of healthcare services, from basic public health services to advanced tertiary treatments. The ethical approval for this study was obtained from the Institutional Review Board, King Abdullah International Medical Research Centre, with Approval Number IRB/2928/23.

**Figure 1:**
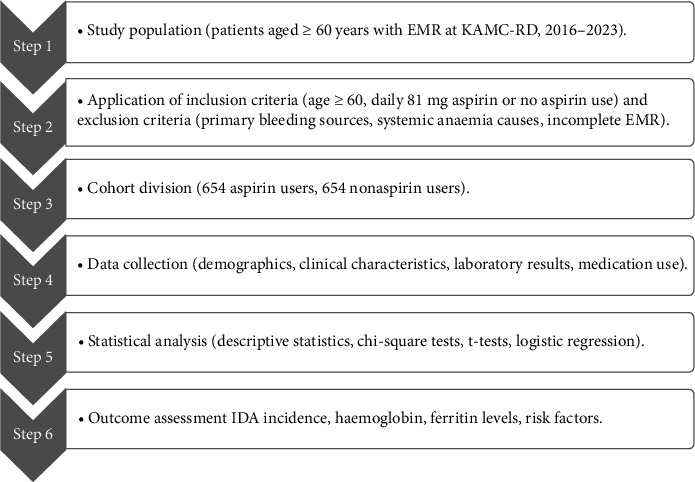
Flowchart of study design and participant selection.

Figure 1 demonstrates the planned retrospective cohort research at the KAMC-RD, Saudi Arabia, for 2016–2023. The study population was patients aged 60 with electronic medical records (EMRs). Inclusion criterion was patients who take aspirin (aspirin user) or no aspirin (nonaspirin user) daily. Exclusion criteria were known primary sources of bleeding (e.g., ulcerative colitis and major surgery), systemic anaemia causes (e.g., chronic kidney disease) or missing EMR. The number of patients sampled was 1308 (654 of whom are using aspirin and 654 are using nonaspirin), determined through a sample size predetermination through a 50% response distribution, a 5% margin of error and a 95% confidence interval. EMR data were extracted regarding demographics, clinical features, laboratory (haemoglobin, ferritin and mean corpuscular volume [MCV]) and medication history. The statistical analyses were descriptive statistics, chi-square tests, independent samples *t*-tests and binary logistic regression.

### 2.2. Study Participants

The selection of the patients was based on specific criteria. Inclusion criteria for the study were patients 60 years and older, patients with EMRs from 2016 to 2023 and patients who had been using daily low-dose aspirin (81 mg) for the aspirin users' group. The exclusion criteria were having a documented history of a primary bleeding source, whether gastrointestinal (e.g., ulcerative colitis) or extragastrointestinal (e.g., recent surgery, particularly primary surgical intervention), or other well-recognized systemic causes of anaemia (e.g., chronic kidney disease) and incomplete EMR. As there was no incidence or prevalence for the study to estimate the sample size, the best approach was to use a response distribution of 50% because that would give enough cases. Using a margin of error of 5% and a confidence interval of 95% and based on the Raosoft sample size calculator, the sample size required was approximately 385 patients.

### 2.3. Data Collection Process

All required data and information were retrieved from the patient's electronic health record (EHR) system at King Abdullah International Medical Research Centre. Trained research assistants conducted the data extraction process, adhering to research ethical considerations and respecting patient privacy. This involved reviewing the files of patients who received daily low-dose aspirin (81 mg) prescriptions (aspirin users' group) and those who did not (nonaspirin users' group) to gather relevant data, including prescription dates and the duration of aspirin use. Additionally, information on patient demographics (age, gender and body mass index [BMI]), the presence or absence of IDA through laboratory reports (including haemoglobin, MCV, ferritin and total iron-binding capacity) and the history of other health conditions (such as congestive heart failure [CHF], HTN and diabetes mellitus [DM]) was collected. The chronic use of other medications, including steroids, anticoagulants, proton-pump inhibitors (PPIs) and nonsteroidal anti-inflammatory drugs (NSAIDs), was also recorded.

Patients are typically diagnosed with IDA based on clinical risk factors associated with low Hb levels (< 130 g/L for men and 120 g/L for women), in addition to microcytic anaemia (MCV < 80). The diagnosis of IDA included Hb levels (< 130 g/L in men and < 120 g/L in women), microcytic anaemia (MCV < 80 fL) and a serum ferritin level less than 21.8 μg/L in men and less than 15 μg/L in women in this study. The male ferritin lower reference range (21.8–274.6 μg/L) was used to set the ferritin cut-off value, and the standard cut-off of 15–20 was maintained in women due to established guidelines [9, 9]. The serum ferritin level below 15 μg/L further supports the diagnosis [9]. We applied the same criteria to diagnose IDA, except for ferritin levels; at KAMC-RD, the reference range for men was 21.8–274.6 μg/L, and for women, it was 4.6–204 μg/L.

### 2.4. Data Analysis

Statistical Package for Social Sciences (SPSS) (v.29.0) (IBM Corp., Armonk, NY, USA) was used to conduct the data analysis. The patient demographics, including age, gender, BMI and duration of aspirin use, were summarized using descriptive statistics, whereas frequencies were used to depict categorical variables. Frequency tables were used to estimate the incidence of IDA for both patient groups, aspirin and nonaspirin users. Cross-tabulation analyses were conducted to test the relationships between the groups and variables, including DM, HTN, CHF, use of steroids, anticoagulants, PPIs, NSAIDs, IDA and gender. The differences in age, gender, BMI and duration of aspirin use between the aspirin and nonaspirin user groups were examined using independent samples tests. Binary logistic regression models of multivariate analysis were used to control for confounders and to determine IDA predictors. Independent samples *t*-tests for equality of means were used to examine variations in ferritin and Hb levels between aspirin and nonaspirin users. A significance level of *p* ≤ 0.05 was considered.

## 3. Results

The patients (*n* = 1308) in this study were divided into two categories regarding daily low-dose aspirin (81 mg) use, i.e., 654 aspirin users and 654 nonaspirin users. The age and the BMI between the groups had a significant difference. Aspirin users had a much older age (72 ± 9 years) compared to nonaspirin users (64 ± 3 years), with a *p* value of less than 0.001. In terms of BMI, the mean BMI of the aspirin users was lower (29.23 + 6.54) than that of the nonaspirin users (30.77 + 6.63) with a *p* value of < 0.001, which shows that the mean BMI of aspirin users and nonaspirin users differs significantly. The study's findings indicate that age and BMI may be confounding variables in the study results that affect the incidence of IDA without the impact of taking aspirin. It is known that age-related physiological differences and BMI differences influence health outcomes, such as iron metabolism and risk of anaemia. Consequently, there is a strong need to consider these issues when deciphering the correlation between the use of aspirin and the occurrence of IDA to have an overall view of the study outcome.

Regarding the clinical characteristics of our study patients, Table 1 compares the distribution of clinical characteristics and medication use in elderly patients (aged ≥ 60 years) in KAMC-RD, Saudi Arabia (2016–2023), between those taking daily low-dose aspirin (81 mg) and those taking no aspirin. The prevalence of DM was not different in groups, which indicates that the miRNA did not confound IDA. Nonaspirin users were at a significant risk of HTN,a this could augment the risk of developing IDA because of the gastrointestinal complications linked to it. CHF did not have any significant difference between groups. Use of steroids, PPIs and NSAIDs was found to be significantly greater in nonaspirin users, which could increase the risk of IDA because of their effects in the gastrointestinal tract. The use of anticoagulants was not different in groups. The IDA prevalence was lower among aspirin users, but not significantly. There were also differences in gender distribution, with more men being the users of aspirin.

Table 2 presents the incidence of IDA in elderly patients (aged ≥ 60 years) at KAMC-RD, Saudi Arabia (2016–2023), stratified by the use of low doses of aspirin (81 mg) per day and nonaspirin. The analysis entails the number and the fraction of patients with and without IDA, the overall sample size, the incidence rate, the *Z*-value and the *p* value of chi-square analysis. The risk of IDA was marginally lower among aspirin users than in nonaspirin users; however, the disparity was not statistically significant, implying that daily low-dose aspirin does not significantly impact the risk of IDA in the group. This observation could be attributed to the multifactorial aetiology of IDA among elderly patients, in which factors such as chronic diseases or dietary deficiencies could contribute to the overshadowing of the role of aspirin in causing IDA through subclinical gastrointestinal bleeding.

Table 3reports the results of independent sample t-tests of Hb (g/L) and ferritin (μg/L) in elderly patients (aged 60 years) with daily, low doses of aspirin (81 mg) and nonaspirin use at the KAMC-RD, Saudi Arabia (2016–2023). The test of equality of variances was carried out using the Levene test, and *t*-tests were used to determine the differences in the means (significance at *p* < 0.05). Aspirin users showed much lower Hb levels than nonaspirin users, indicating a relation between aspirin and subclinical bleeding or iron loss in the gastrointestinal tract. Conversely, ferritin levels were much more elevated in aspirin users, which may be associated with the role of ferritin as an acute-phase reactant in the case of inflammation or comorbidity, regardless of reducing haemoglobin. These results suggest that the use of aspirin can be linked to a change in haematological parameters. However, it does not always imply that the incidence of IDA could increase.

Table 4 shows the multivariate logistic regression results to determine the effects of demographic and clinical factors on the risk of IDA among older patients (aged [?] 60 years) at the KAMC-RD, Saudi Arabia (2016–2023), using demographic and clinical variables and stratified by daily low-dose aspirin intake (81 mg) and no aspirin intake. The variables will be age, gender, BMI, DM, HTN, CHF, steroids, anticoagulants, PPIs and NSAIDs. In aspirin users, HTN was slightly correlated with higher risks of IDA, indicating that it may also be connected with gastrointestinal complications aggravated by aspirin. Age, gender, BMI, DM, CHF, steroids, anticoagulants, PPIs and NSAIDs did not have a significant influence on IDA in this group. In nonaspirin users, age, being female, having DM and CHF and using steroids were significantly related to increased risk of developing IDA, probably due to age-related changes in iron absorption, gender-specific prevalence of anaemia, inflammation by chronic diseases and gastrointestinal alterations caused by steroid use. Anticoagulants, NSAIDs, BMI and HTN were not significantly associated with IDA in nonaspirin users. This implies that these factors have minimal impact on the group's IDA risk.

Table 5 presents the time taken by elderly patients (aged [?] 60 years) at KAMC-RD, Saudi Arabia (2016–2023), to use low-dose aspirin (81 mg) per day based on the presence or absence of IDA and the sexes (men and women) with IDA. Independent sample *t*-tests were used to compare the mean length of stay, which was reported as standard deviations, confidence intervals, *t*-values and *p* values. The period of aspirin intake between IDA patients and those who did not have the disease showed no significant difference between the two groups, and this indicates that the duration of aspirin exposure will not play an important role in the development of IDA in this group. Likewise, there were no significant differences in the time of aspirin use in men and women with IDA, which shows that gender does not alter the relationship between the duration of aspirin use and IDA risk. These results have indicated that comorbidities or dietary factors and not the period of aspirin use may influence the risk of IDA more in elderly aspirin users.

## 4. Discussion

The study was a retrospective cohort study that was conducted at KAMC-RD, Saudi Arabia (2016–2023), aimed to explore the relationship between daily low-dose aspirin (81 mg) use and the development of IDA among the elderly (60 years and above), involving 654 aspirin users and 654 nonusers. The findings offer nuanced insights into the complex interaction between aspirin consumption, blood parameters and clinical risk factors, which may contribute to current discussions on the safety of low-dose aspirin in older adults. In response to the reviewer's request for a well-structured discussion that transforms raw data into meaningful insights, this section interprets the study results, contextualizes them within existing literature and considers their clinical and research implications, including relevant sources.

This major observation that there was no statistically significant difference between IDA occurrence in aspirin users (20.8) and nonaspirin users (25.2) refutes the currently existing hypothesis that low-dose aspirin significantly raises the risk of IDA by causing gastrointestinal bleeding [7, 8]. This nonsignificance with a minor *p* value (0.057) indicates that the antiplatelet effects of aspirin, which have been linked to the risk of gastrointestinal haemorrhage, may not be a significant cause of IDA in this group [7]. This observation could be attributed to several factors. Age effects on gastrointestinal iron absorption, chronic illnesses and polypharmacy are the predisposing factors to multifactorial anaemia in elderly patients, which may mask the role of aspirin in the incidence of IDA [10]. As an example, HTN, steroid use, PPIs and NSAIDs were more prevalent in nonaspirin users in this study, all of which are related to gastrointestinal irritation or bleeding, which may predispose IDA regardless of aspirin [11]. These competing risk factors could have contributed to the slightly increased IDA in nonaspirin users, which would counteract the anticipated effect of aspirin-induced bleeding.

One of the key outcomes was the reduced Hb levels in the aspirin users over the nonaspirin users, despite the insignificant differences in the incidence of IDA. This observation is consistent with previous studies, such as a randomized controlled trial (ASPREE), which found higher anaemia rates and Hb decreases among healthy older people taking low doses of aspirin, although without evident bleeding [7]. Hb reductions with low-dose aspirin use were also reported in another study of older men with acute coronary syndrome or ischaemic stroke. They were attributed to subclinical gastrointestinal blood loss or other mechanisms [12]. A 2024 review also hypothesized that aspirin metabolites, including 2,3-dihydroxybenzoic acid (2,3-DHB) and salicyluric acid, can chelate iron, which leads to its excretion and contributes to Hb losses without necessarily being manifested as overt IDA [13]. This process might be behind the reported Hb decrease in aspirin users, with a small but significant effect on erythropoiesis or iron homeostasis that may not necessarily become diagnostic of IDA.

On the contrary, the surprise discovery that higher levels of ferritin were found in the case of aspirin users makes it difficult to understand their iron status. Ferritin is also a reactant in the acute phase and an indicator of iron stores, which are also being raised in the inflammatory conditions, including but not limited to HTN, DM or other comorbid conditions prevalent in the aspirin user population [10, 13]. The present investigation reported an increased mean age (72 vs. 64 years) and a statistical tendency of HTN and IDA risk among aspirin users, which is that chronic inflammation might be the primary cause of ferritin rise, concealing the body iron deficiency [14]. Reduced use of PPI in patients taking aspirin could have allowed subclinical bleeding of the gastrointestinal tract, which decreased the Hb with inflammation, maintaining consistent ferritin levels, creating a paradoxical haematological picture [15]. This discovery highlights the weakness of using ferritin alone in diagnosing IDA in older patients with comorbidities because inflammatory conditions may distort its measurements [14]. Newer research would include more biomarkers, including hepcidin or transferrin saturation, to differentiate between iron deficiency and inflammation-induced ferritin increases in a better way [15, 15].

The multivariate logistic regression analysis (Table 4) showed that the unique IDA risk factors differed in groups. Only HTN was marginally correlated with heightened risk of IDA in aspirin users, which implies that aspirin might enhance endothelial damage or gastrointestinal susceptibility caused by HTN in previous research [7, 16]. The lack of substantial connections with age, gender, BMI, DM, CHF, steroids, anticoagulants, PPIs or NSAIDs in this group suggests that the effect of aspirin on IDA could be context-dependent, which is influenced by risk factors. Nonaspirin users, however, had substantial predictors of IDA, and these were advanced age, female sex, DM, CHF and steroid use. These results are consistent with the existing literature reporting the effects of ageing on iron absorption, anaemia being more prevalent among women because of hormonal or physiological reasons and inflammation caused by chronic diseases as altering iron metabolism [14, 15, 17]. The risk of IDA was probably increased by steroid use, which has been known to result in gastrointestinal irritation, which would have increased mucosal damage, as is the case with nonaspirin users [16]. The absence among nonaspirin users of the significant associations with BMI, HTN, anticoagulants, PPIs or NSAIDs indicates that these factors do not play an important independent role in the occurrence of IDA in nonaspirin users.

The duration of aspirin use (Table 5) showed no significant differences between patients with and without IDA. Also, it showed no differences between men and women with IDA, which indicates that the duration of aspirin use is not a significant factor in determining the risk of IDA. This observation differs from the expectations that there can be increased gastrointestinal bleeding and iron loss when the use of aspirin is prolonged [7]. Instead, other variables, including dietary iron or comorbidities or comedications, might have a stronger role in developing IDA [13, 18]. The significant disparity in aspirin use between men and women (a greater proportion of aspirin users are men) could be due to prescribing by cardiovascular preventive influences. However, the fact that aspirin users exhibited no gender-specific differences in risk of IDA indicates that aspirin does not have a strong gender-dependent haematological effect [15].

These findings have several clinical implications. The significant difference between the Hb in aspirin users and those with a stable incidence of IDA needs to be highlighted as a reminder of the importance of routine haematology monitoring in geriatric patients receiving low-dose aspirin, especially with HTN, to identify early anaemia or iron deficiency [14, 19]. Clinicians ought to ponder coprescription of PPIs to reduce the risk of gastrointestinal bleeding because the reduced intake of PPI by aspirin consumers could have been a cause of Hb deterioration [11]. The fact that ferritin concentrations in the users of aspirin are high indicates the need to interpret ferritin in the backdrop of inflammation, and it may be necessary to conduct further tests, such as C-reactive protein or hepcidin, to determine the iron status [10, 15]. In nonaspirin users, it is possible to note that strong relations between IDA and age, female gender, DM, CHF and steroids allow focussing on the screening of high-risk populations, and the management of underlying chronic diseases and the reduction in steroid-based gastrointestinal effects should be emphasized [14, 16].

This study's strengths are its large sample size, strong statistical analysis to control various confounders and a clearly defined elderly cohort, which strengthens the validity of the study findings in the context. Nevertheless, restrictions are to be taken into consideration. The retrospective design restricts causal inference and can cause bias in selection, mainly since the method relies on electronic medical history records, which are likely to be distorted by documentation errors. Unmeasured confounders such as iron intake in food, socioeconomic status or physical activity may affect the risk of IDA [13, 18]. In the single-centre setting, these conditions might diminish generalizability to other areas, populations or healthcare systems with varied prescribing practices or patient demographics.

Prospective and multicentre designs should be used in future studies to create causality and improve external validity. The molecular pathways of the iron-chelating metabolites of aspirin and how they affect iron homeostasis might help elucidate the observed Hb and ferritin differences [13, 15]. Also, the multifactorial aetiology of IDA in the elderly might be more precisely defined by studies involving a combination of nutritional assessments and inflammatory biomarkers [26, 28]. The study of the effectiveness of preventive interventions, including PPI coprescription or iron supplementation, might inform individualized treatment strategies to offset the cardiovascular versus the haematological risks of aspirin use [11, 14].

In this research, critical observations of the immediate haematological impact of low-dose aspirin in individuals of advanced age are raised, pointing to reduced Hb levels and increased ferritin levels without a massive rise in the incidence of IDA. The study improves the risk–benefit consideration of aspirin in getting cardiovascular preventive advantages, and the significance of personalized monitoring and management plans to optimize the results in this high-risk group is highlighted.

### 4.1. Limitations and Strengths of the Study

The strong design with a large sample size of 1308 elderly patients (654 aspirin users and 654 nonaspirin users) is what gives this retrospective cohort study a significant advantage as it has a sufficient statistical strength to identify differences in the incidence of iron deficiency anaemia (IDA) and factors that can predispose a patient to it, which increases the validity of the research outcomes in the context of the study. The combination of extensive statistical testing, including descriptive statistics, chi-square tests, independent samples *t*-tests and binary logistic regression, allowed us to conduct a complete assessment of predictors of IDA without previous effects caused by the influence of other factors, including age, gender, BMI and comorbidities. A tertiary care centre was used to extract data stored in EMRs, which guaranteed both the thoroughness of information (demographics, clinical characteristics, laboratory results [haemoglobin, ferritin, MCV] and medication history) and ethical control and respect for patient privacy by well-trained research assistants. The clinically relevant question that the study has focused on, which was to evaluate the haematological impacts of low-dose aspirin in a high-risk elderly group of patients, provides a viable implication in clinical monitoring and management, as approved by the Institutional Review Board. Nevertheless, the retrospective nature has weaknesses such as selection bias, and causal inference is impossible as it uses past data. The use of EMR is prone to miscategorized or incomplete records, which could lead to inaccurate diagnoses of IDA and the estimation of variables. Although the study has accounted for various confounders, the issue is that unmeasured variables such as nutritional iron, socioeconomic status or physical activity could affect the outcome. The small-scale environment of KAMC-RD restricts the possibility of generalization to a vast population or healthcare system with different prescribing patterns or disease rates. Also, local ferritin reference ranges, which are different from international ones, can be used, and thus, this can affect the comparability of IDA classification. These advantages and drawbacks should be considered while interpreting the study results and developing future studies.

## 5. Conclusion

A retrospective cohort study of 1308 elderly patients aged 60 years and older, both daily low-dose aspirin (81 mg) users and nonusers in KAMC-RD, Saudi Arabia, conducted in 2016–2023, does not show significant differences in incidence of IDA in both groups (20.8 and 25.2 cases, respectively). Conversely, aspirin users were found to have considerably lower Hb concentration and elevated ferritin, indicating evidence of subclinical iron loss or inflammatory effects, which may not always lead to acute IDA. HTN also became a marginal risk factor of IDA among aspirin users, which may worsen gastrointestinal vulnerability. Among nonaspirin users, the age of the patients, women, DM, CHF, chronic steroids and DM were strong predictors of IDA, indicating the multifactorial aspect of anaemia in this category of patients. Age and BMI were also cited as possible confounding factors, so there is a strong necessity to be alert to monitor haematological parameters of the elderly taking aspirin. These results help to narrow the risk–benefit analysis of low-dose aspirin in cardiovascular disease prevention. They would recommend individual approaches, including routine Hb and ferritin testing, particularly in hypertensive individuals. In future studies, emphasis must be put on prospective, multicentre studies to explain the long-term effects of aspirin on iron metabolism, pathophysiology and intervention in different population groups to maximize therapeutic effects.

## Figures and Tables

**Table 1:** Distribution of health conditions and medication use among aspirin users and nonaspirin users.

Property	Aspirin users *N* (%)	Nonaspirin users *N* (%)	*χ* ^2^	*p* value
*Diabetes mellitus*				
No	264 (49.8%)	266 (50.2%)	0.013	0.910
Yes	390 (50.1%)	388 (49.9%)		

*Hypertension*				
No	236 (54.5%)	197 (45.5%)	5.251	0.022
Yes	418 (47.8%)	457 (52.2%)		

*Congestive heart failure*				
No	564 (49.3%)	579 (50.7%)	1.560	0.212
Yes	90 (54.5%)	75 (45.5%)		

*Steroids*				
No	602 (51.6%)	565 (48.4%)	10.882	< 0.001
Yes	52 (36.9%)	89 (63.1%)		

*Anticoagulants*				
No	576 (49.8%)	581 (50.2%)	0.187	0.665
Yes	78 (51.7%)	73 (48.3%)		

*Proton-pump inhibitors*				
No	556 (52.1%)	511 (47.9%)	10.300	0.001
Yes	98 (40.7%)	143 (59.3%)		

*Nonsteroidal anti-inflammatory drugs*				
No	573 (52.4%)	520 (47.6%)	15.635	< 0.001
Yes	81 (37.7%)	134 (62.3%)		

*Iron deficiency anaemia*				
No	518 (51.4%)	489 (48.6%)	3.629	0.057
Yes	136 (45.2%)	165 (54.8%)		

*Gender*				

**Table 2:** Association between daily low-dose aspirin use and iron deficiency anaemia (IDA) incidence.

Distribution	Iron deficiency anaemia (no)	Iron deficiency anaemia (yes)	Total *N*	Incidence rate	*Z*-value	*p* value
Aspirin users	518 (79.2%)	136 (20.8%)	654	207.95	−1.905	0.057
Nonaspirin users	489 (74.8%)	165 (25.2%)	654	252.29	—	—

**Table 3:** Comparison of haemoglobin (Hb, g/L) and ferritin (μg/L) levels between aspirin users and nonaspirin users.

**Distribution**		**Levene's test for equality of variances**		***t*-Test for equality of means**						
		***F***	**Sig.**	***t*-Values**	**df**	**Sig. (two-tailed)**	**Mean difference**	**Std. error difference**	**95% confidence interval of the difference**	
									**Lower upper**	

Haemoglobin level (g/L)	Equal variances assumed	26.409	0.000	−8.108	43,799	0.000	−1.809598	0.223180	−2.247036	−1.372161
	Equal variances not assumed			−8.098	43,088.011	0.000	−1.809598	0.223474	−2.247612	−1.371585

Ferritin level (μg/L)	Equal variances assumed	92.528	0.000	8.459	9103	0.000	270.115127	31.933580	207.518138	332.712116
	Equal variances not assumed			7.882	5173.641	0.000	270.115127	34.268942	202.933518	337.296736

Abbreviations: BMI = body mass index, Hb = haemoglobin, IDA = iron deficiency anaemia and MCV = mean corpuscular volume.

**Table 4:** Binary logistic regression model assessing iron deficiency anaemia (IDA) predictors among aspirin and nonaspirin users.

Factor	*B*	S.E.	Wald	df	Sig.	Exp (*B*)
*Aspirin users*						
Age	0.018	0.012	2.275	1	0.131	1.018
Gender (women)	0.352	0.208	2.846	1	0.092	1.421
BMI	−0.010	0.016	0.390	1	0.532	0.990
Diabetes mellitus (yes)	0.256	0.226	1.280	1	0.258	1.292
Hypertension (yes)	0.462	0.240	3.712	1	0.054	1.587
Congestive heart failure (yes)	−0.088	0.285	0.095	1	0.758	0.916
Steroids (yes)	0.916	0.491	3.483	1	0.062	2.499
Anticoagulants (yes)	0.678	0.538	1.591	1	0.207	1.971
Proton-pump inhibitors (yes)	−0.810	0.637	1.618	1	0.203	0.445
NSAIDs (yes)	−0.341	0.585	0.340	1	0.560	0.711
Constant	−2.950	1.012	8.505	1	0.004	0.052

*Nonaspirin users*						
Age	0.071	0.033	4.690	1	0.030	1.074
Gender (women)	0.535	0.210	6.506	1	0.011	1.708
BMI	−0.026	0.016	2.891	1	0.089	0.974
Diabetes mellitus (yes)	0.645	0.210	9.433	1	0.002	1.907
Hypertension (yes)	0.324	0.229	2.005	1	0.157	1.383
Congestive heart failure (yes)	0.716	0.266	7.237	1	0.007	2.046
Steroids (yes)	0.916	0.450	4.150	1	0.042	2.500
Anticoagulants (yes)	0.279	0.397	0.495	1	0.482	1.322
Proton-pump inhibitors (yes)	−0.874	0.588	2.209	1	0.137	0.417
NSAIDs (yes)	0.030	0.480	0.004	1	0.950	1.030
Constant	−5.898	2.169	7.394	1	0.007	0.003

**Table 5:** Duration of aspirin use concerning iron deficiency anaemia (IDA) status and gender.

	Mean ± SD	95% confidence interval	*t*-value	*p* value
*Iron deficiency anaemia*				
No	198 ± 296	−63.168, 49.434	−0.240	0.811
Yes	205 ± 299			

*Gender*				
Men	180 ± 286	−152.687, 50.463	−0.990	0.321
Women	231 ± 313			

## Data Availability

The datasets used in and/or analysed in the current study are available from the responding author upon reasonable request.

## References

[1] World Health Organization (2008). *World Health Organization Worldwide Prevalence of Anaemia 1993–2005: WHO Global Database on Anaemia*.

[2] Smith D. L. (2009). Anemia in the Elderly. *Iron Disorders Institute Guide to Anemia*.

[3] Culleton B. F., Manns B. J., Zhang J., Tonelli M., Klarenbach S., Hemmelgarn B. R. (2006). Impact of Anemia on Hospitalization and Mortality in Older Adults. *Blood*.

[4] Penninx B. W., Pahor M., Woodman R. C., Guralnik J. M. (2006). Anemia in Old Age Is Associated With Increased Mortality and Hospitalization. *The Journals of Gerontology Series A: Biological Sciences and Medical Sciences*.

[5] Zakai N. A., Katz R., Hirsch C. (2005). A Prospective Study of Anemia Status, Hemoglobin Concentration, and Mortality in an Elderly Cohort: The Cardiovascular Health Study. *Archives of Internal Medicine*.

[6] Gaskell H., Derry S., Andrew Moore R., McQuay H. J. (2008). Prevalence of Anaemia in Older Persons: Systematic Review. *BMC Geriatrics*.

[7] Mahady S. E., Margolis K. L., Chan A. (2021). Major GI Bleeding in Older Persons Using Aspirin: Incidence and Risk Factors in the ASPREE Randomised Controlled Trial. *Gut*.

[8] Gaskell H., Derry S., Moore R. A. (2010). Is There an Association Between Low Dose Aspirin and Anemia (Without Overt Bleeding)? Narrative Review. *BMC Geriatrics*.

[9] Camaschella C. (2015). Iron Deficiency: New Insights Into Diagnosis and Treatment. *Hematology*.

[10] Chey W. D., Woods K. L., Scheiman J. M. (2019). Proton Pump Inhibitors and Aspirin: A Review of Clinically Important Drug Interactions. *American Journal of Therapeutics*.

[11] Theurl I., Mairhofer K., Stauder R. (2019). Anemia in the Elderly: Not Always What It Seems. *Hämostaseologie*.

[12] Raizada M. S., Raizada M. S., Raina S., Sharma R., Yadav R. S. (2022). Association Between Use of Low-Dose Aspirin on Hemoglobin Levels and Serum Iron Homeostasis in Patients With Acute Coronary Syndrome or Ischemic Stroke. *International Journal of Noncommunicable Diseases*.

[13] Sandnes M., Ulvik R. J., Vorland M., Reikvam H. (2021). Hyperferritinemia: A Clinical Overview. *Journal of Clinical Medicine*.

[14] Lopez A., Cacoub P., Macdougall I. C., Peyrin-Biroulet L. (2016). Iron Deficiency Anaemia. *Lancet*.

[15] Thomas E. J., Burstin H. R., O’Neil A. C., Orav E., Brennan T. A. (1996). Patient Noncompliance With Medical Advice After the Emergency Department Visit. *Annals of Emergency Medicine*.

[16] Fertrin K. Y. (2020). Diagnosis and Management of Iron Deficiency in Chronic Inflammatory Conditions (CIC): Is Too Little Iron Making Your Patient Sick?. *Hematology*.

[17] Stauder R., Valent P., Theurl I. (2018). Anemia at Older Age: Etiologies, Clinical Implications, and Management. *Blood*.

[18] Nemeth E., Ganz T. (2006). Regulation of Iron Metabolism by Hepcidin. *Annual Review of Nutrition*.

[19] Goodnough L. T., Schrier S. L. (2014). Evaluation and Management of Anemia in the Elderly. *American Journal of Hematology*.

[20] Fletcher A., Forbes A., Svenson N., Wayne Thomas D. (2022). Guideline for the Laboratory Diagnosis of Iron Deficiency in Adults (Excluding Pregnancy) and Children. *British Journal of Haematology*.

[21] Vanasse G. J., Berliner N. (2010). Anemia in Elderly Patients: An Emerging Problem for the 21st Century. *Hematology 2010, the American Society of Hematology Education Program Book 2010*.

[22] Randi M. L., Bertozzi I., Santarossa C. (2020). Prevalence and Causes of Anemia in Hospitalized Patients: Impact on Diseases Outcome. *Journal of Clinical Medicine*.

[23] Wratsangka R., Putri R. A. N. H. (2020). The Importance of Anemia and Health-Related Quality of Life in the Elderly. *Universa Medicina*.

